# Time-to-positivity in bloodstream infection for *Candida* species as a prognostic marker for mortality

**DOI:** 10.1093/mmy/myad028

**Published:** 2023-04-05

**Authors:** Caitlin Keighley, Alun L Pope, Debbie Marriott, Sharon C-A Chen, Monica A Slavin, Australia and New Zealand Mycoses Interest Group, Ian Arthur, Rob Baird, Narin Bak, Christopher Blyth, Jeannie Botes, Belinda Chapman, Elaine Cheong, Louise Cooley, Kathryn Daveson, Rob George, Emma Goeman, Krispin Hajkowicz, Catriona Halliday, Christopher H Heath, Ann Hofmeyr, Pankaja Kalukottege, Alison Kesson, Karina Kennedy, Sarah Kidd, Tony M Korman, Michael Leung, Eunice Liu, Nenad Macesic, Kyle McDonald, Brendan McMullan, Orla Morrissey, Stella Pendle, Jenny Robson, Tania Sorrell, Neil Underwood, Sebastian van Hal, Kerry Weeks, Heather Wilson

**Affiliations:** Centre for Infectious Diseases and Microbiology Laboratory Services, Institute of Clinical Pathology and Medical Research, New South Wales Health Pathology, Westmead Hospital, Sydney, NSW 2145, Australia; University of Sydney Institute for Infectious Diseases, Westmead, Sydney, NSW 2156, Australia; Southern.IML Pathology, Sonic Healthcare, Coniston NSW 2500, Australia; Eastern Health Clinical School, Monash University, Melbourne, Victoria 3124, Australia; Analytical Insight, Crawley 6009, WA, Australia; Department of Microbiology and Infectious Diseases, St. Vincent's Hospital, Sydney 2010, NSW, Australia; Centre for Infectious Diseases and Microbiology Laboratory Services, Institute of Clinical Pathology and Medical Research, New South Wales Health Pathology, Westmead Hospital, Sydney, NSW 2145, Australia; University of Sydney Institute for Infectious Diseases, Westmead, Sydney, NSW 2156, Australia; Department of Infectious Diseases, Peter MacCallum Cancer Centre, National Centre for Infections in Cancer, Melbourne, VIC 3052, Australia

**Keywords:** candidaemia, time to positivity, mortality, candida, bloodstream infection

## Abstract

Time-to-positivity (TTP) may assist in predicting the outcome of candidaemia. We analysed a candidaemia dataset collected prospectively in Australia over 1 year (2014–2015). TTP was defined as the period from blood culture sampling to the blood culture flagging positive. Of 415 candidaemia episodes, overall, 30-day mortality was 29% (120/415); mortality with *Candida albicans* was 35% (59/169), *C. glabrata* complex, 37% (43/115), *C. tropicalis*, 43% (10/23), *Pichia kudriavzevii* 25% (3/12), and *C. parapsilosis* complex 7% (5/71). Each day of increased TTP multiplied the odds ratio (OR) of survival at 30 days by a factor of 1.32 [95% confidence interval (CI) 1.06–1.69]. Shorter TTP was associated with increased mortality, with 1-day TTP associated with 30-day mortality 37% (41/112) (95%CI: 28%–46%) and 5-day TTP 11% (2/18) (95%CI: 2%–36%).

Time-to-positivity (TTP) for *Candida* spp. in blood cultures is a parameter that may help predict the prognosis of bloodstream infection (BSI) with *Candida* (candidaemia). A higher organism load might be expected in the blood of patients with a more serious disease and thus, a shorter TTP in blood cultures.^1^ Indeed with few exceptions,^2,3^ the majority of data support an association of shorter TTP with higher mortality in BSIs^4–7^ including candidaemia in BSI.^8^ Species also affect TTP, with *C. glabrata* complex associated with a longer TTP than other species.^9–11^ Two recent studies counterintuitively reported higher mortality with longer TTP in candidaemia but included small numbers in one and a focus on paediatric cases with *C. parapsilosis* complex candidaemia and marked antifungal resistance in the other.^3,9^ Species-specific differences are evident, with e.g. *C. parapsilosis* complex associated with a lower mortality.^12^ Thus, analysis of the effect of TTP should be species-specific.

We performed a prospective multicentre study of 527 episodes of candidaemia in Australia between 2014–2015.^13,14^ Resistance was uncommon, as previously reported.^13^ Risk factors for increased 30-day mortality in 133 cases with extended clinical data included age, source of infection, and intensive care unit (ICU) admission; causative species and choice of antifungal therapy were not associated with mortality.^14^ Here, we present data available from a larger dataset examining TTP (time from blood culture sampling to the blood culture flagging positive) and mortality.

We analysed an Australian candidaemia dataset in which information on TTP and mortality had been collected but not previously reported.^13^ Laboratory surveillance was conducted for a period of 12 months from the time of enrolment at 29 sites in 2014–2015.^13^ Study approval was granted by human research ethics committees (HREC reference: AU RED LNR14/WMEAD/112).

Candidaemia was defined as isolation of *Candida* spp. from blood cultures. Episodes involving more than one *Candida* species [all mixed infections included *C. albicans*, additional species included *C. glabrata* complex (*n* = 7), *C. parapsilosis* complex (*n* = 1), *C. lipolytica* (*n* = 1), and *C. dubliniensis* (*n* = 1)] and those with data that was incomplete to 30 days (*n* = 102) were excluded. For clinical relevance, ‘*Candida’* species refer to all *Candida* spp. as well as those now re-classified as belonging to closely-related yeast genera e.g. *Candida krusei* (now *Pichia kudriavzevii*).^15^

Species identification was confirmed at two reference laboratories with matrix-assisted laser desorption/ionization time-of-flight mass spectrometry (MALDI–TOF MS) (Biotyper database v3.1; Bruker Daltoniks, Germany) and internal transcribed spacer rDNA sequencing as required. Logistic regression analysis with survival at 30 days as the response, and TTP, *Candida* species, patient age, and gender as explanatory variables was used to assess the effect of TTP. We performed a Kaplan–Meier analysis with TTP (in whole days) as strata, and a separate analysis with species as strata. Analyses were performed using R 20.5.0.^16^

There were 415 episodes with TTP recorded and mortality data to 30 days. The median age of subjects was 62 years (interquartile range [IQR] 48–74). The overall all-cause 30-day mortality was 29% (125/415). Causative species included *Candida albicans* (*n* = 169), *Candida glabrata* complex (*n* = 114), *Candida parapsilosis* complex (*n* = 72), *Candida tropicalis* (*n* = 23), *Pichia kudriavzevii* (*n* = 12), *Clavispora lusitaniae* (formerly *Candida lusitaniae, n* = 10)*, Candida dubliniensis* (*n* = 8)*, Yarrowia lipolytica* (formerly *Candida lipolytica, n = 3)*, and one each of *Candida colliculosa, Kluyveromyces marxianus* (previously *Candida kefyr*)*, Candida quercitrusa*, and *Candida robusta*.

For all episodes, the median TTP was 48 h (IQR 33–65 h). Kaplan–Meier survival curve estimates are shown in Figure 1. These demonstrate that shorter TTP was associated with increased mortality with the species-dependent association. Species-specific candidaemia mortality is in Table 1. The mortality for *C. parapsilosis* complex candidaemia was 3-6 fold lower at 7% (5/72). The significance of reduced mortality with *C. parapsilosis* complex was confirmed by the log-rank (Mantel–Haenszel) test (*χ*^2^ statistic = 25.2 on 5 degrees of freedom, *P* < .001).

**Figure 1.**
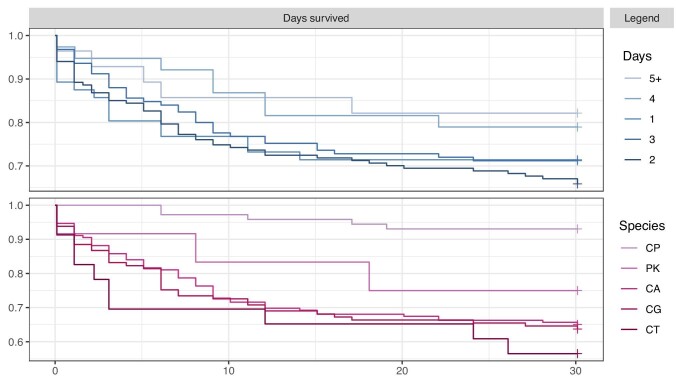
Kaplan–Meier survival curve estimates by TTP and Candida species. Vertical axis is survival probability and horizontal axis is number of days survived (censored at 30 days). In the legend, entries are ordered by survival probability at 30 days, ‘Days’ refers to TTP in days, and ‘Species’ is coded as follows: CP, *Candida parapsilosis* complex; PK, *Pichia kudriavzevii*; CA, *Candida albicans*; CG, *Candida glabrata* complex; CT, *Candida tropicalis*.

**Table 1.** Mortality and TTP by species of candidaemia.

	30-day mortality % (n/n)	95% confidence interval for 30-day mortality	Hours TTP Median (IQR)
*C. albicans*	35% (59/169)	28%–43%	49 (37–69)
*C. glabrata* complex	37% (42/114)	28%–46%	55 (39–72)
*C. parapsilosis* complex	7% (5/72)	3%–16%	48 (36–59)
*C. tropicalis*	43% (10/23)	24%–65%	27 (21–42)
*P. kudriavzevii*	25% (3/12)	7%–57%	33 (24–54)

95% confidence intervals were calculated by standard binomial approximations.

TTP: time to positivity; IQR: interquartile range.

Logistic regression modelling indicated that TTP, patient age, and species had significant effects on mortality. One day reduction of TTP multiplied the odds ratio (OR) of mortality at 30 days by a factor of 1.32 (95%CI 1.06–1.69). A year less of age multiplied the OR of mortality at 30 days by a factor of 0.96 (95%CI 0.94–0.97). To understand the magnitudes of these effects, these numbers imply that a day less of TTP worsened the estimated 30-day mortality by approximately the same amount as 7 years of additional age increased it. A TTP of 1 day was associated with a 30-day mortality of 36% vs 11% with a TTP of 5 days (Fig. 1).

In addition, the logistic regression model had species as an explanatory variable, and the estimated effects for *C. albicans, C. glabrata* complex, *C. tropicalis*, and *P. kudriavzevii* were all significant in comparison with *C. parapsilosis* complex as the reference. The effects for these species were to multiply the OR for mortality by between 5.26 and 11.11 in comparison with *C. parapsilosis* complex. A TTP effect was demonstrated for the subset consisting of all cases of non-*C. parapsilosis* complex infection. No TTP effect was shown in the subset consisting of *C. parapsilosis* complex cases.

The analysis we present has harnessed a large dataset of candidemia episodes (*n* = 415) to assess the relationship between TTP in blood cultures and mortality. It corroborates previous findings suggesting that shorter TTP is associated with increased mortality where 152 episodes of candidaemia were analysed.^8^ Our findings are not consistent with those of a recent study.^3^ It is possible that the results of Hamilton et al.^3^ of a TTP effect in the opposite direction is a chance result, especially given the small number of patients with candidaemia (*n* = 53). It is also of note that the mortality of candidaemia of 47.2% in their data is higher than recent literature or the data presented here of 30-day mortality of ∼30%.^17,18^

Strengths of the present analysis include the prospective and multicentre nature of the data collection, allowing more uniform data collection and encompassing broad representation from Australian laboratories. However, there are a number of limitations of the study. Firstly, the time from blood culture collection to commencement of incubation, and the location of where the patients received their care, were not recorded. It is possible that blood cultures collected from patients located outside metropolitan areas had a longer transport time, and once incubation was commenced, a shorter time to positivity. In 23/26 study sites the candidaemia episodes in our dataset were from laboratories on-site at tertiary hospitals and where the TTP may more closely reflect the organism load; there was no effect observed between study site and TTP. Secondly, the number of individual species available for subgroup analysis was limited, and this data should be interpreted with caution. Thirdly, whilst bias was minimized as individual cases were counted even if they died on the same day as the blood culture was collected, we cannot rule out that patients would be less likely to have blood cultures collected on the day they passed away. Finally, clinical variables including co-morbidities, data on prolonged blood culture positivity and treatment were not available in this dataset.

Our findings are consistent with what is biologically plausible, i.e. that a large inoculum in the bloodstream will cause both a shorter time to positivity and poorer outcome. This is, however, but one of many variables associated with the outcome of candidaemia, including source, patient factors, and *Candida* species.^12,14^ Further research examining candidaemia should include an assessment of TTP by species as well as clinical variables.

In summary, our analysis supports the conclusion that a shorter TTP is associated with increased mortality in candidaemia, with species-specific differences influencing TTP.
